# Transparent Graphene Interfaces for Capacitive Recordings from hiPSC-Derived Cardiomyocyte Monolayers: A Proof-of-Concept Study

**DOI:** 10.3390/s26144383

**Published:** 2026-07-10

**Authors:** Melanie Meincke, Andre Bazzone, Sonja Stoelzle-Feix, Stephan Holzhauser, Maria Barthmes, Lars Richter, Izabela Kamińska, Michael George, Philip Tinnefeld, Niels Fertig

**Affiliations:** 1 Nanion Technologies, Ganghoferstraße 70A, 80339 Munich, Germany; 2 Department of Chemistry, Ludwig-Maximilians-Universität, Butenandtstraße 5-13, 81377 Munich, Germany

**Keywords:** biosensor, cardiomyocytes, dofetilide, graphene, capacitive sensing, cell–sensor interface

## Abstract

**Highlights:**

**What are the main findings?**

- Transparent monolayer graphene can be integrated as a conductive sensing interface in a capacitive recording platform for hiPSC-derived cardiomyocyte monolayer analysis.
- Graphene-based sensors enabled optical pre-assessment of monolayer integrity and synchronous beating, while capacitive transients could be recorded from analyzable hiPSC-CM monolayer preparations.

**What are the implications of the main findings?**

- Transparent conductive interfaces may support workflows that combine optical inspection of cell-layer quality with label-free functional electrophysiological readout.
- The results establish a methodological proof of concept for graphene-based capacitive cardiomyocyte recordings and motivate further studies on reproducibility, scalability, and cell–sensor interface effects.

**Abstract:**

Transparent conductive interfaces can enable optical pre-assessment of cardiac cell layers while remaining compatible with label-free electrophysiological recording. Here, we evaluated the integration of a monolayer graphene electrode into a capacitive recording platform for the analysis of human-induced pluripotent stem cell-derived cardiomyocyte (hiPSC-CM) monolayers. hiPSC-CMs cultured on graphene sensors formed confluent, synchronously beating monolayers that could be assessed by light microscopy prior to recording. Capacitive current transients could be recorded from spontaneously beating hiPSC-CM monolayers, supporting the compatibility of transparent graphene interfaces with capacitive recordings from electrically active cardiac cell layers. Signal amplitude and waveform morphology varied across sensors, indicating that recording performance depended strongly on the cell–sensor interface, including cell attachment, monolayer integrity, and capacitive coupling at the sensing surface. A descriptive perturbation sequence using the hERG blocker dofetilide revealed changes in waveform morphology and beat timing across sequential recordings. However, the data do not allow firm attribution to a compound-specific effect and are not intended for quantitative pharmacological characterization. Overall, the results support graphene as a transparent conductive cell–sensor interface, which should be interpreted in the context of cell–substrate interactions at the sensing surface. Combining optical pre-assessment with functional capacitive readout may support integrated workflows. Further studies will be needed to differentiate material-, interface-, and recording-related contributions and to establish reliable conditions for reproducible and scalable recordings.

## 1. Introduction

Accurate monitoring of cardiomyocyte electrophysiology in vitro is a central component of drug development and cardiac safety assessment [1,2,3,4]. Reliable detection of repolarization disturbances remains critical, as delayed repolarization and early afterdepolarization-like events are closely associated with proarrhythmic risk [3].

Human-induced pluripotent stem cell-derived cardiomyocytes (hiPSC-CMs) are widely used as a model system in preclinical compound screening to capture such electrophysiological dynamics in a human-relevant context [2,4,5,6,7]. Beyond classical voltage-based extracellular recordings, capacitive electrophysiology platforms have been established as an alternative approach for detecting electrogenic activity in excitable cells [8].

In these systems, rapid changes in transmembrane charge distribution induce transient displacement currents at the cell-electrode interface, which are recorded without direct electrical contact or voltage clamping. Capacitive recording platforms therefore provide a suitable framework for label-free recordings from electrogenic cell systems. In standard configurations, capacitive coupling is established via planar metallic electrodes, typically based on gold. Gold provides reliable electrical conductivity and compatibility with established microfabrication processes, and thiol-based surface chemistry is widely used for gold surface functionalization [9]. However, opaque metallic sensing surfaces limit direct microscopic assessment of the active cell layer before recording. In cardiomyocyte assays, where signal quality depends strongly on the formation of a confluent and synchronously beating monolayer, this can hinder identification of preparations suitable for electrophysiological analysis. A transparent sensing interface could therefore provide a practical advantage by enabling optical pre-assessment of cell coverage, morphology, and coordinated contraction on the active sensing area prior to recording.

Transparent conductive materials such as graphene and indium tin oxide (ITO) provide possible routes toward such interfaces [10,11,12]. Among transparent conductive materials, graphene is particularly attractive for cell-based sensing because it combines optical transparency with high electrical conductivity, high charge-carrier mobility, an atomically thin two-dimensional structure, and reported compatibility with cellular adhesion and viability [13,14,15,16,17]. In addition, graphene-based sensor architectures can be implemented in different electronic readout configurations, including solution-gated field-effect transistor measurements and capacitive recordings [15,16,18]. The primary motivation for evaluating graphene in the present study was not an expected gain in recording performance relative to alternative materials, but the possibility of optically assessing hiPSC-CM monolayers cultured on the active sensing area while also evaluating whether capacitive signals can be recorded through the same transparent conductive cell–sensor interface.

Here, we introduce a methodological adaptation of an established capacitive recording approach by replacing the conventional thiol-functionalized gold sensing surface with a transparent monolayer graphene electrode while preserving the underlying capacitive measurement principle. This approach enables direct culture of hiPSC-CMs on the sensing interface, optical assessment of monolayer integrity and synchronous beating prior to electrical recording. Using dofetilide as an illustrative perturbation, we examined whether graphene-enabled capacitive recordings from hiPSC-CM monolayers can reflect changes in the recorded transients while allowing prior optical pre-assessment of the same sensing surface. The study was therefore designed as a proof-of-concept evaluation of a transparent cell–sensor interface rather than to validate the platform for pharmacological screening or long-term cardiomyocyte culture.

## 2. Results

### 2.1. Graphene-Based Capacitive Recordings of hiPSC-CMs

**Figure 1:**
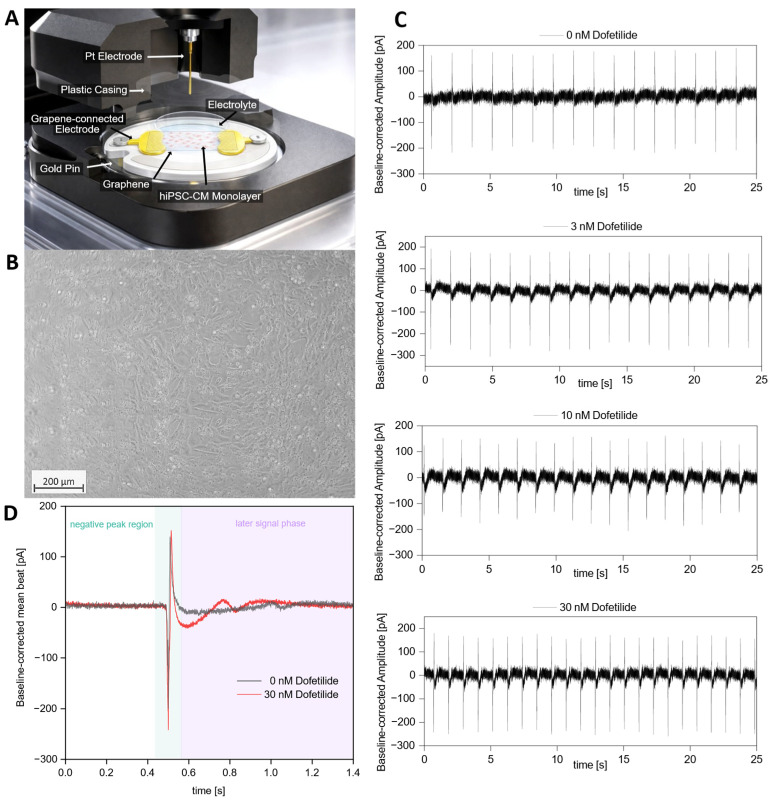
Graphene-enabled capacitive recordings from hiPSC-CMs. (**A**) Schematic cross-section of the graphene-based sensor architecture used in the present capacitive recording configuration. The underlying graphene sensor architecture and fabrication process were described previously for solution-gated field-effect transistor operation [18]. Here, the graphene-connected electrode, contacted from below via a single gold pin, served as a transparent planar sensing electrode. hiPSC-CMs were cultured directly on the graphene surface, and a platinum electrode was immersed in the electrolyte from above without contacting the cell layer. Capacitive signals were recorded between the graphene-connected bottom electrode and the platinum electrode. (**B**) Representative bright-field image of hiPSC-CMs cultured on the transparent graphene sensing area prior to capacitive recording. The image represents a still frame from the corresponding bright-field recording shown in Supplementary Video S1, in which coordinated contraction of the cell layer allows the synchronously beating hiPSC-CM monolayer to be distinguished from the background. (**C**) Representative baseline-corrected capacitive current transients recorded using the adapted capacitive recording system from hiPSC-CMs cultured on graphene sensors under control conditions (0 nM) and after exposure to increasing concentrations of dofetilide (3, 10, and 30 nM). The traces illustrate descriptive differences in the recorded capacitive transients across the representative sequential series. (**D**) Overlay of baseline-corrected mean-beat waveforms under control conditions and after dofetilide treatment. Operational annotations indicate the negative peak region and the later signal phase. The overlay shows a difference in the later signal phase of the representative mean-beat trace.

Graphene-based sensor substrates integrated into the adapted capacitive recording system enabled capacitive recordings of spontaneously beating hiPSC-CMs. A schematic overview of the recording configuration and a representative microscopic image of cardiomyocytes cultured on the graphene sensing surface are shown in Figure 1A,B. The cardiomyocytes formed a confluent, synchronously contracting cell layer on the transparent electrode within three days of culture. The optical transparency of the graphene layer enabled direct microscopic inspection of cell coverage, morphology, and coordinated contraction prior to electrical recordings (Supplementary Video S1).

### 2.2. Baseline Reproducibility Across Independent Graphene-Based Sensors

**Figure 2:**
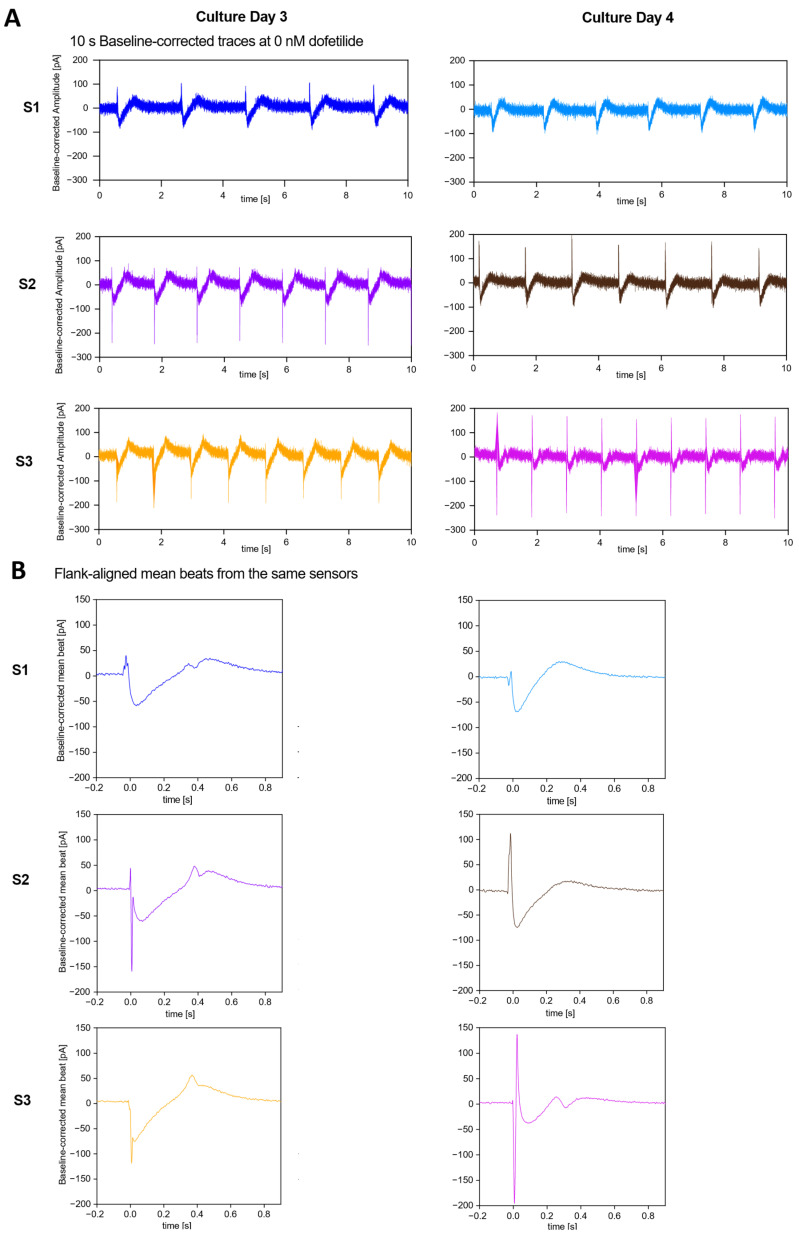
Baseline capacitive recordings across independent graphene-based sensors. (**A**) Baseline-corrected current traces recorded at 0 nM dofetilide from six independent graphene-based sensors. Three sensors were analyzed on culture day 3 and three additional sensors on culture day 4. S1–S3 denote individual sensors within the respective culture day. Capacitive transients were detected in all analyzed sensors, while signal amplitude and waveform morphology varied between preparations. (**B**) Corresponding flank-aligned mean-beat traces calculated for each sensor. Waveform shape and signal amplitude depended on the individual cell–sensor interface. These results demonstrate that recurring, alignable capacitive transients were obtained across independent graphene-based sensors.

To address reproducibility of the graphene-based capacitive recording configuration, additional baseline recordings were analyzed from independent graphene-based sensors. In total, six independent sensors were included in the baseline analysis, with three sensors measured on culture day 3 and three additional sensors measured on culture day 4. For each sensor, baseline recordings of 25 s duration were acquired at 0 nM dofetilide. The complete 25 s traces are provided in Supplementary Figure S1, while representative 10 s segments are shown in Figure 2A. Recurrent capacitive transients were detected in all analyzed sensors, demonstrating that the observation of activity-associated capacitive signals was not limited to a single representative recording.

At the same time, signal amplitude, waveform morphology, and signal clarity varied between sensors. This variability is consistent with the interpretation that the recorded capacitive signal depends strongly on the individual cell–sensor interface, including monolayer coverage, cell attachment, contractile synchronization, graphene integrity, and capacitive coupling. To compare the recurring waveform morphology within each sensor, flank-aligned mean-beat waveforms were calculated separately for each 25 s baseline recording. Recurrent capacitive transients within each trace were aligned to the descending flank of the negative capacitive transient rather than to the absolute negative peak minimum, thereby reducing the influence of narrow peak-like artifacts on the averaged waveform (Figure 2B). Mean beats were not averaged across sensors because waveform shape and signal amplitude differed between independent preparations. The analysis therefore supports reproducibility of the general recording principle across independent graphene-based sensors, while also highlighting preparation-dependent variability in signal morphology.

For additional comparison, capacitive transients from a gold-based sensor and a graphene-based sensor were analyzed under 0 nM dofetilide conditions (Supplementary Figure S2). For each sensor type, three consecutive 10 s recordings were evaluated. Recurrent capacitive transients were observed for both the gold- and the graphene-based sensor, indicating that activity-associated capacitive signals could be detected with the same general capacitive recording principle on both sensing surfaces. The corresponding flank-aligned mean-beat waveforms calculated from the three consecutive recordings showed distinct waveform morphologies for gold and graphene. This comparison was performed descriptively and was not intended as a quantitative performance comparison between electrode materials because the two sensor types differ in surface chemistry, optical accessibility, and cell–sensor interface formation. Rather, the comparison supports the interpretation that the recorded transients reflect an interface-coupled capacitive signal, while the practical advantage of graphene in the present context lies in optical accessibility of the active sensing area.

### 2.3. Descriptive Perturbation Sequence Using Dofetilide

Descriptive baseline-corrected capacitive current traces recorded under control conditions and after stepwise exposure to dofetilide are shown in Figure 1C. IKr/hERG-blocking compounds such as dofetilide, E-4031, and cisapride are commonly used as reference compounds during the establishment of hiPSC-CM-based recording platforms [4,5]. In the present study, dofetilide was included as an illustrative perturbation to assess whether the recording configuration could detect changes in the recorded interface transients. The experiment was not designed as a full assay calibration, a dose–response study, or a quantitative pharmacological validation of the platform. Under control conditions (0 nM), capacitive transients were similar in shape across consecutive beats, exhibiting an initial fast deflection, a pronounced negative peak region, and a later signal phase. Exposure to increasing concentrations of dofetilide (3, 10, and 30 nM) was associated with alterations in the recorded capacitive traces across the representative concentration series. These affected the initial fast deflection, the negative peak region, and the later signal phase. As the capacitive recordings reflect an integrated response at the cell-electrode interface, these signal features were not assigned to specific ionic currents or action-potential phases. Overlay of temporally aligned and amplitude-normalized beats (Figure 1D) further highlighted a shift in the later signal phase under dofetilide exposure while minimizing differences in absolute timing and baseline offsets.

### 2.4. Quantitative Analysis of Beat Timing and Negative Peak Amplitude

To provide a descriptive summary of beat timing and negative peak amplitude within the representative concentration series, inter-spike intervals (ISI) and baseline-corrected negative peak amplitudes were determined from the recurrent capacitive transients detected within the respective 25 s recording windows (Table 1). Mean ISI decreased across the sequential recordings from 1524 ms under control conditions to 1097 ms at 30 nM dofetilide, indicating shorter intervals between consecutive negative peaks. Beat timing remained highly regular in all analyzed trace segments, as reflected by low ISI coefficients of variation below 1%. A modest increase in ISI CV was observed at 30 nM, but this did not indicate pronounced beat-to-beat irregularity in the analyzed 25 s window. Negative peak amplitudes varied across the sequential recordings without a monotonic concentration-dependent pattern. The recorded transients showed descriptive changes across the sequential measurement series. However, because similar time-dependent changes may occur in the absence of compound application and waveform appearance depended on signal processing, the present data do not allow firm attribution of these changes to a compound-specific effect.

Taken together, the sequential recordings show descriptive changes in capacitive transients and beat timing across the representative series. However, the present data do not allow firm attribution of these changes to a compound-specific effect and are therefore intended as a proof-of-concept illustration rather than as a quantitative pharmacological characterization.

## 3. Discussion

In this study, we show that a transparent monolayer graphene electrode can be integrated into an established capacitive recording architecture and can support functional recordings from hiPSC-CM monolayers. The main contribution of the present work is not the validation of a pharmacological assay, but the demonstration that graphene can function as a transparent and conductive cell–sensor interface that enables optical pre-assessment of monolayer integrity before electrical measurement. Within this methodological proof-of-concept setting, baseline recordings from six independent graphene-based sensors across two culture days showed recurrent capacitive transients, demonstrating that signal detection was not limited to a single representative recording. In addition, one sensor with clearly resolved and stable baseline waveform morphology was used for a representative dofetilide perturbation sequence. Our findings support the interpretation that the recorded transients arise from an interface-coupled capacitive signal shaped by the cell layer, sensing surface, coating, and recording configuration, rather than from a direct measurement of membrane potential.

The present work should therefore be understood as an initial feasibility study rather than as a full validation of a cardiomyocyte assay platform. A comprehensive validation would require systematic comparison with established culture substrates and electrode materials, additional readouts such as calcium imaging or molecular markers of cell state, longer culture periods with assessment of cell viability, monolayer integrity, synchronous contractile activity, capacitive signal quality over time, and pharmacological dose–response experiments. These experiments were beyond the scope of the present proof-of-concept study, but they represent important next steps for evaluating the broader applicability of graphene-based capacitive cardiomyocyte recordings.

The capacitive recordings shown in Figure 2 demonstrate that recurrent capacitive signal detection was not restricted to a single representative recording. Recordings from six sensors measured on culture days 3 and 4 showed recurring transients which were suitable for beat-timing and flank-aligned mean-beat analysis. These data support the reproducibility of the general graphene-based capacitive recording principle. At the same time, the recordings revealed clear sensor-to-sensor variability in signal amplitude and waveform morphology. This variability indicates that successful recordings depend not only on the presence of a conductive graphene interface, but also on the biological and physical quality of the cell–sensor interface.

The observation that only a subset of prepared graphene-based sensors yielded analyzable recordings further highlights the importance of the cell–sensor interface. Potential contributing factors include graphene integrity after transfer, electrical contact quality, surface cleanliness, cell attachment, monolayer coverage, contractile synchronization, and the degree of capacitive coupling between the cell layer and the sensing electrode. Future optimization should therefore address both sensor fabrication and the biological interface, including more standardized surface preparation, controlled cell seeding and culture conditions, systematic optical quality criteria prior to recording, and larger datasets across independently prepared sensors.

Sequential recordings obtained after dofetilide addition were analyzed from the sensor that showed the clearest and most stable baseline waveform morphology among the independent baseline recordings. This dataset was used as a representative perturbation sequence to illustrate whether waveform changes could be followed after stepwise compound exposure. The recordings showed descriptive changes in waveform shape, negative peak amplitude, and beat timing across the representative series. However, because similar time-dependent changes may occur in the absence of compound application, the present dataset does not allow firm attribution of these changes to a compound-specific effect. The dofetilide series should therefore be interpreted as an illustrative perturbation used to assess whether activity-associated changes in the recorded interface transients could be detected, rather than as evidence of a specific pharmacological response or as a calibration of the graphene-based capacitive recording configuration. Accordingly, the present experiment does not establish pharmacological accuracy, assay robustness, or a dose-dependent dynamic range of the graphene-based capacitive recording configuration. A full validation of the system for cardiac electrophysiology applications would require concentration-response experiments with multiple well-characterized reference compounds. In particular, compounds producing distinct and ideally opposing effects, such as isoproterenol and verapamil, would be important to determine whether the system can reliably capture dose-dependent changes in beating rate and waveform dynamics in both directions. Because the present recordings represent an integrated capacitive response at the cell-electrode interface, the individual signal features were not assigned to defined ionic currents or discrete action-potential phases. Instead, they were interpreted descriptively as synchronized monolayer activity and changes in capacitive waveform dynamics. In contrast to intracellular action-potential recordings or field-potential measurements, the capacitive waveform is shaped not only by cellular electrophysiological activity but also by cell–substrate coupling, surface chemistry, monolayer morphology, and the electrical properties of the sensing interface. Parameters such as rise time, decay kinetics, or later waveform components may therefore be useful descriptive metrics in future studies, but their interpretation as specific depolarization or repolarization phases would require validation against alternative readouts such as patch-clamp, field-potential, impedance, or calcium imaging measurements. Mechanistic clarification would further require targeted pharmacological dissection using channel-specific blockers and clearer separation of electrophysiological and mechanically coupled signal contributions.

The main methodological advance of the present approach lies in establishing graphene as a transparent alternative to conventional opaque sensing surfaces while combining functional capacitive readout with optical accessibility. Graphene’s transparent interface enables direct inspection of cell coverage, monolayer integrity, and synchronous contractile behavior on the active sensing area before recording. This is relevant during assay establishment, where variation in cell attachment or syncytium formation can strongly affect signal quality. In the present configuration, the main practical advantage of graphene was therefore optical accessibility of the active sensing area rather than a demonstrated gain in recording sensitivity, maturation, or pharmacological performance. This optical accessibility enables pre-assessment of monolayer integrity and coordinated beating before recording and may therefore support more informed selection of suitable preparations. The variability observed across the six independently analyzed recordings further underscores the relevance of cell–sensor interface conditions for recording performance and highlights optical pre-assessment as a useful workflow component. In this sense, graphene is not simply an alternative electrode material but supports a more informative workflow by linking optical assessment of the cell layer with functional capacitive recording.

Several limitations need to be considered. First, the present study remains a methodological proof of concept based on a limited number of independently analyzed sensors. Although six independent graphene-based sensors yielded analyzable baseline recordings and were included in the reproducibility analysis, the dataset is not sufficient for statistical evaluation or for establishing assay robustness. In addition, the complete dofetilide concentration sequence was analyzed in detail from one representative sensor selected based on baseline signal morphology and signal stability. The resulting values should therefore be interpreted as a descriptive perturbation analysis rather than as measurements across independent biological replicates or as a statistically powered concentration-response experiment. Second, a direct comparison with conventional gold-based sensors was not feasible under the exact culture conditions used in this study. The available gold sensors were pre-functionalized with thiol-based surface chemistries optimized for capacitive coupling in solid-supported membrane measurements. These surface modifications are not primarily designed for cardiomyocyte adhesion. Consequently, stable hiPSC-CM monolayers could not be reproducibly established on these substrates under the conditions applied here. Differences in cell attachment and monolayer formation can therefore not be attributed to the electrode material alone, but likely reflect the combined effects of electrode material, surface chemistry, active area, and cell–sensor interface formation.

The exploratory gold/graphene comparison shown in Supplementary Figure S2 provides additional support for the interpretation that the observed transients reflect a general interface-coupled capacitive recording principle rather than a graphene-specific artifact. Sequential baseline recordings from both a gold-based and a graphene-based sensor showed recurrent capacitive transients, while the corresponding flank-aligned mean-beat waveforms displayed distinct waveform morphologies. These differences should be interpreted cautiously, because gold and graphene differ in surface chemistry, electrode/electrolyte interface properties, optical accessibility, and cell–sensor coupling conditions. The present data do not allow the waveform differences or polarity characteristics to be assigned to a specific material property of graphene alone. Therefore, the comparison is not interpreted as evidence for superior recording performance of either material. Instead, it supports the view that capacitive cardiomyocyte signals can be detected with the general recording principle on both sensing surfaces, whereas the principal advantage of graphene in the present configuration lies in optical accessibility of the active sensing area.

Alternative transparent conductive materials such as indium tin oxide (ITO) were not evaluated in the present study. Therefore, the current data do not allow a direct comparison between graphene, ITO, and other transparent conductive substrates. Graphene is used here as a proof-of-concept material for implementing a transparent conductive cell–sensor interface, rather than as a material demonstrated to be superior to other transparent conductive electrode materials. Moreover, the present dataset did not include repeated controls following the same stepwise application protocol, so time-dependent changes or handling-related effects cannot be fully excluded. The recordings also do not distinguish between purely electrophysiological signal components and possible mechanically coupled contributions associated with contraction.

Finally, the biological interpretation is limited by the fact that the capacitive signal represents a cluster measurement rather than a direct readout from a defined ion channel population. At the same time, this network-level character can also be seen as an advantage since the recorded response reflects coordinated activity of a cardiomyocyte layer rather than the behavior of an isolated single cell. The present data should therefore be regarded as a proof-of-concept study of a transparent cell–sensor interface rather than as a robust or fully characterized assay platform. The present work defines a feasible transparent interface concept and a practical workflow for preselection of suitable monolayers before recording.

Taken together, the present results support further evaluation of transparent conductive interfaces for cardiomyocyte recordings, with graphene serving here as a proof-of-concept material. The analyzed dataset demonstrates that recurrent capacitive transients can be detected across independent graphene-based sensors, while the representative dofetilide sequence illustrates that waveform changes can be followed within a sequential perturbation experiment. Future work should clarify how material properties, cell–sensor interface formation, and recording conditions contribute to signal quality, reproducibility, and scalability, and should determine how optical pre-assessment can be integrated into more standardized recording workflows.

The present results suggest several useful directions for further development. These include testing additional reference compounds with different electrophysiological modes of action, comparing the approach directly with established methods such as impedance-, field potential-, or calcium-based assays, and investigating whether graphene-based sensor fabrication offers practical advantages in material usage, consumable design, and manufacturing scalability.

It will also be important to better understand the different culture behavior on graphene and thiolated gold surfaces and to improve reproducibility across sensors. In the longer term, the transparent graphene-based approach may prove useful for workflows that combine optical pre-assessment of monolayer quality with label-free electrophysiological recording and for applications that require closer correlation between cell layer integrity and functional readout.

## 4. Materials and Methods

### 4.1. Cell Culture

Cardiosight-S hiPSC-CMs (NEXEL Co., Ltd., Seoul, Republic of Korea) were cultured on graphene sensor substrates based on the Cardiosight-S User Guide with assay-specific optimization. Prior to cell seeding, graphene sensor wells were cleaned with isopropanol and ddH_2_O and coated with 50 µL fibronectin solution in sterile PBS for at least 1 h at 37 °C. Although the manufacturer recommends a 1:20 fibronectin working dilution, a 1:100 dilution was used here based on internal experience with these cells and sensor surfaces. Cells were thawed carefully according to the supplier’s protocol, centrifuged at 180× *g* for 3 min, resuspended in plating medium, and seeded at 50,000 cells per sensor in a final plating volume of 200 µL.

The first medium change was performed after 24 h by replacing 100 µL plating medium with 100 µL maintenance medium. Thereafter, daily half-medium changes were performed. Recordings were carried out on day 3 because longer culture periods led to cell aggregation and reduced suitability for stable capacitive recording. The optical transparency of the graphene layer enabled direct inspection of beating behavior, monolayer integrity, and surface coverage prior to electrical recordings. Bright-field images were acquired on the same sensor shortly before capacitive recording using a separate inverted transmitted-light microscope (Primovert, Carl Zeiss Microscopy GmbH, Jena, Germany). Optical imaging was not performed simultaneously with electrical measurements.

### 4.2. Graphene Sensor Architecture and Capacitive Measurement Configuration

Capacitive recordings were performed using a commercial capacitive recording system (SURFE^2^R, Nanion Technologies, Munich, Germany) equipped with a graphene-based sensor (Figure 1A). The sensor consists of a planar electrode structure integrated into a plastic carrier forming a defined measurement well that is filled with electrolyte solution. The manufacturing process of the sensors was established in previous work [18]. In the previously described solution-gated field-effect transistor configuration, source and drain gold contacts were connected by the graphene monolayer, while a platinum electrode immersed in the electrolyte served as the liquid gate. In that configuration, the measurement chamber was equipped with two bottom-side contact pins for contacting the source and drain gold electrodes. In the present study, however, the graphene-based sensor was not operated in transistor mode. Instead, it was placed in the capacitive measurement chamber of the established capacitive readout system, which contacts one graphene-connected bottom electrode from below via a single gold pin. The continuous graphene film connected to this contact was therefore used as a transparent planar bottom sensing electrode for capacitive readout. The capacitive signal was recorded between the graphene-connected bottom contact and the platinum electrode immersed in the electrolyte.

Cardiosight-S hiPSC-CMs were cultured directly on the graphene surface, forming the active cell-electrode interface. During measurements, a platinum electrode was immersed in the electrolyte from above without contacting the cell layer. Electrogenic activity of the cardiomyocytes induces transient displacement currents across the cell-electrode interface, which are detected capacitively between the graphene sensing surface and the platinum electrode. The recorded signal reflects transient currents arising from time-dependent changes in charge distribution at the cell-electrode interface. These currents are converted into a measurable voltage signal by the recording electronics via current-to-voltage amplification. The resulting waveforms therefore represent interface-coupled charging processes shaped by the sensing surface and its coating rather than direct measurements of membrane potential. Apart from the sensing interface and associated sensor layout, all other components of the capacitive recording system were kept identical to the standard gold-based configuration.

### 4.3. Compound Application and Recording Conditions

Dofetilide was applied manually by half-medium exchange to minimize mechanical disturbance of the cardiomyocyte layer. For each concentration step, 100 µL of medium was carefully added to an existing 100 µL volume, resulting in a final assay volume of 200 µL and the intended final compound concentration. Mixing was achieved by gentle dispensing followed by one careful pipetting step at the well edge. In practice, the addition step was performed over approximately 5 s, and the next recording was started after 10 s according to a fixed timing protocol. This procedure was chosen because complete solution exchange frequently disrupted monolayer integrity and promoted contraction into localized syncytial clusters. Dofetilide was applied gradually at final concentrations of 0, 3, 10, and 30 nM. For each concentration, two consecutive capacitive traces of 25 s duration each were recorded from the same sensor, separated by a brief equilibration period to confirm signal stability and to exclude relevant drift between successive measurements. All recordings were performed at 37 °C using a temperature-controlled recording chamber. Control experiments with the respective DMSO dilutions were performed on separate sensors. Under these conditions, separate control recordings did not show obvious changes in beat number, inter-spike interval, or waveform shape within the analyzed traces. However, because these controls were not part of the same sequential series, time-dependent changes and handling-related effects cannot be fully excluded.

### 4.4. Signal Processing and Quantitative Analysis

Recorded capacitive traces were extracted from the software of the capacitive recording system and further processed in OriginPro 2019 (OriginLab Corporation, Northampton, MA, USA). For waveform inspection, unfiltered exported traces were used to avoid filter-induced smoothing of fast signal components. Baseline drift was corrected in OriginPro using the Peak Analyzer tool. For each trace, baseline anchor points were identified in regions between capacitive transients using the second-derivative zero method, followed by manual adjustment where required. A baseline was then generated by linear interpolation between the selected anchor points and subtracted from the raw trace to compensate for baseline drift. This correction was applied only to remove slow baseline offsets and was not intended to alter beat timing, peak position, or waveform morphology.

A total of 10 graphene-based sensors were prepared and microscopically assessed. Prior optical evaluation on the transparent sensor surface was used to identify sensors bearing intact, synchronously beating monolayers and to exclude preparations with incomplete coverage or impaired syncytium formation from electrophysiological analysis. Six independent sensors yielded analyzable baseline recordings and were included in the reproducibility analysis shown in Figure 2 and Supplementary Figure S1.

For the reproducibility analysis shown in Figure 2, recordings at 0 nM dofetilide were analyzed from six independent graphene-based sensors. Three sensors were measured on culture day 3 and three additional sensors on culture day 4. For each sensor, a 25 s baseline-corrected recording window was used for beat detection, beat-timing analysis, and mean-beat analysis. Representative 10 s segments are shown in Figure 2A, whereas the complete 25 s traces are provided in Supplementary Figure S1.

Capacitive transients within each 25 s trace were identified based on the recurring negative transient region. Inter-spike intervals (ISI) were defined as the time difference between consecutive negative transient events and are reported as mean ± standard deviation (SD). Beat-to-beat timing regularity was assessed using the coefficient of variation in the inter-spike interval (ISI CV), calculated as the ratio of the ISI standard deviation to the mean ISI. The resulting descriptive baseline parameters for the six independent sensors are summarized in Supplementary Table S1.

Flank-aligned mean-beat waveforms were evaluated separately for each 25 s baseline recording. For this analysis, recurrent capacitive transients within each trace were aligned to the descending flank of the negative transient rather than to the absolute negative peak minimum. This alignment strategy was used to compare recurring waveform morphology while reducing the influence of narrow peak-like artifacts or single-point extrema on the averaged waveform. Mean beats were not pooled across sensors because signal amplitude and waveform morphology varied between independent preparations. For the exploratory gold/graphene comparison shown in Supplementary Figure S2, three consecutive 10 s baseline recordings from one gold-based sensor and one graphene-based sensor were analyzed analogously. Capacitive transients detected across the three consecutive recordings were included in the corresponding flank-aligned mean-beat analysis for each sensor type.

For the representative dofetilide perturbation sequence shown in Figure 1 and Table 1, the sensor was selected after comparison of the 0 nM baseline recordings across the independent graphene-based sensors. The selected sensor showed the most clearly resolved and stable waveform morphology, including recurrent capacitive transients, a well-defined negative deflection, and sufficient signal stability for following waveform changes across the subsequent concentration sequence. The sequential recordings from this sensor were therefore used as the representative perturbation dataset for dofetilide exposure at 0, 3, 10, and 30 nM. This analysis was intended as a descriptive perturbation experiment and not as a concentration-response analysis across independent sensors.

All recurrent capacitive transients detected within the respective 25 s recording window were included in the descriptive analysis. The analyzed transients displayed a clearly discernible negative deflection, which enabled consistent extraction of beat timing and negative peak amplitude. Beat parameters were averaged within each concentration condition. *n* refers to the number of analyzed capacitive transients within the respective 25 s recording window, not to independent sensor replicates. Negative peak amplitude was defined as the peak current amplitude relative to baseline for each included transient and is reported as mean ± SD in picoampere (pA).

No inferential statistical comparison across independent sensor replicates was performed because the present study was designed as a methodological proof-of-concept. The expanded baseline dataset was used to assess reproducibility of capacitive signal detection across independent graphene-based sensors, whereas the dofetilide sequence was used as a representative perturbation experiment and not as a quantitative pharmacological validation.

## Figures and Tables

**Table 1:** Descriptive analysis of beat variability and negative peak amplitude in response to dofetilide. Inter-spike intervals (ISI) and baseline-corrected negative peak amplitudes were quantified from capacitive traces recorded over 25 s windows from hiPSC-CMs cultured on graphene-based sensors. ISI values are reported as mean ± standard deviation (SD) in milliseconds, with the coefficient of variation (CV) indicating beat-to-beat regularity. Peak amplitude refers to the mean baseline-corrected negative peak amplitude and is reported in picoampere (pA). Values were extracted from baseline-corrected traces. All recurrent capacitive transients detected within the respective 25 s recording window were included in the analysis. The analyzed transients displayed a clearly discernible negative deflection, enabling consistent extraction of beat timing and negative peak amplitude. Parameters were averaged for each condition. *n* denotes the number of analyzed beats, not independent sensor replicates. The table provides a descriptive summary of the representative traces shown in Figure 1.

Dofetilide (nM)	*n*	ISI Mean ± SD (ms)	ISI CV (%)	Negative Peak Amplitude Mean ± SD (pA)
0	16	1524 ± 3.9	0.26	−(200 ± 12.6)
3	17	1475 ± 2.5	0.17	−(265 ± 15.7)
10	18	1383 ± 6.6	0.48	−(159 ± 22.1)
30	23	1097 ± 9.2	0.84	−(242 ± 8.6)

## Data Availability

The data presented in this study are available from the authors upon request.

## Supplementary Materials

The following supporting information can be downloaded at: https://www.mdpi.com/article/10.3390/s26144383/s1, Video S1: Representative bright-field recording of synchronously beating hiPSC-CMs cultured on a transparent graphene sensor substrate. Figure S1: Complete 25 s baseline capacitive recordings across independent graphene-based sensors. Table S1: Descriptive baseline parameters across independent graphene-based sensors. Figure S2: Comparison of capacitive recordings obtained from gold-based and graphene-based sensors.
