# Supplementary material for: Transparent Graphene Interfaces for Capacitive Recordings from hiPSC-Derived Cardiomyocyte Monolayers: A Proof-of-Concept Study
Source: Sensors (Basel). 2026 Jul 10;26(14):4383. doi: 10.3390/s26144383 (PMC13431396; doi:10.3390/s26144383)
Supplement: Supplementary file 1 [file sensors-26-04383-s001.zip › Supplementary Materials.pdf]

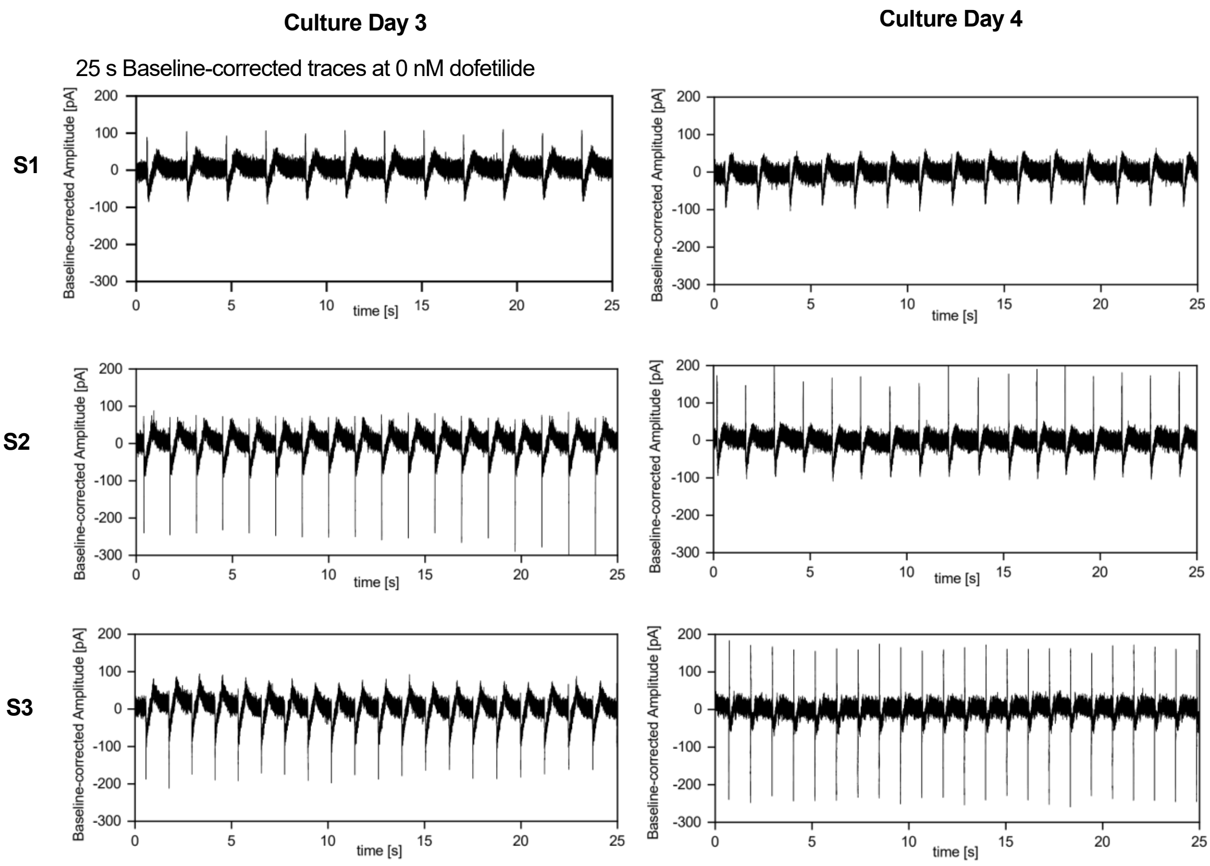

Supplementary Figure S1. Complete 25 s baseline capacitive recordings across independent graphene-based sensors. Baseline-corrected capacitive traces recorded at 0 nM dofetilide from six independent graphene-based sensors are shown. Three sensors were measured on culture day 3 (left) and three additional sensors on culture day 4 (right). S1–S3 denote independent sensors within each culture day. The 10 s segments shown in **Figure 2A** were selected from these complete 25 s recordings. Recurrent capacitive transients were detected in all analyzed sensors over the complete recording window. Signal amplitude and waveform morphology varied between preparations. The same recordings were used for descriptive beat-timing analysis and for the flank-aligned mean-beat waveforms shown in **Figure 2B**.

Supplementary Table S1. Descriptive baseline parameters across independent graphene-based sensors. Inter-spike intervals (ISI) were quantified from 25 s baseline recordings at 0 nM dofetilide. ISI values are reported as mean ± standard deviation (SD), with the coefficient of variation (CV) indicating beat-to-beat regularity. n denotes the number of analyzed capacitive transients within the 25 s recording window, not independent biological replicates. Negative peak amplitude refers to the negative peak of the flank-aligned mean-beat waveform and is reported in picoampere (pA).

Sensor ID	Culture day	n	ISI mean ± SD (ms)	ISI CV (%)	Negative peak amplitude (pA)
D3-S1	3	13	1921 ± 4.9	0.25	−63
D3-S2	3	19	1278 ± 8.5	0.67	−160
D3-S3	3	21	1130 ± 18.3	1.62	−119

D4-S1	4	16	1568 ± 18.4	1.17	−73
D4-S2	4	17	1384 ± 28.7	2.07	−79
D4-S3	4	24	1015 ± 8.7	0.86	−196

Note: D3 and D4 indicate culture day 3 and culture day 4; S1–S3 denote independent sensors measured on the respective culture day.

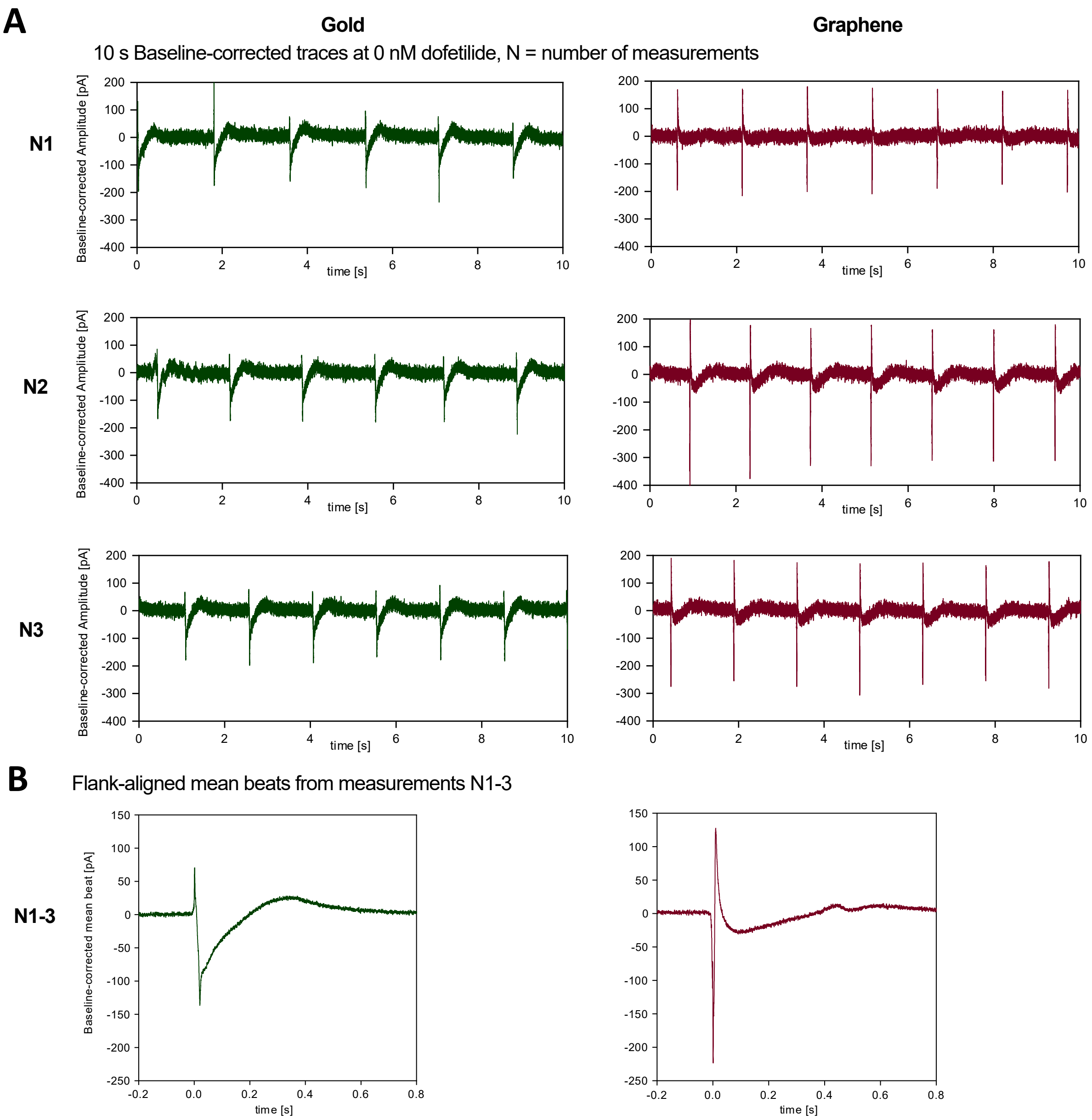

Supplementary Figure S2. Comparison of capacitive recordings obtained from gold-based and graphene-based sensors. **(A)** Baseline-corrected capacitive traces recorded at 0 nM dofetilide from one gold-based sensor and one graphene-based sensor. Three consecutive 10 s recordings (N1–N3) are shown for each sensor type. Recurrent capacitive transients were observed for both sensor types. Waveform morphology and the dominant deflection pattern differed between gold- and graphene-based recordings. **(B)** Flank-aligned mean-beat waveforms calculated from the three consecutive recordings shown in **(A)**. The data are intended as a descriptive comparison supporting the general capacitive recording principle on both sensor types, rather than a quantitative performance comparison between electrode materials.
